# Patient Selection Approaches in FGFR Inhibitor Trials—Many Paths to the Same End?

**DOI:** 10.3390/cells11193180

**Published:** 2022-10-10

**Authors:** Peter Ellinghaus, Daniel Neureiter, Hendrik Nogai, Sebastian Stintzing, Matthias Ocker

**Affiliations:** 1 Global Clinical Development Oncology, Merck Healthcare KGaA, 64293 Darmstadt, Germany; 2 Institute of Pathology, University Clinics Salzburg, Paracelsus Medical University, 5020 Salzburg, Austria; 3 Cancer Cluster Salzburg, 5020 Salzburg, Austria; 4 Ryvu Therapeutics, 30-394 Krakow, Poland; 5 Division of Hematology, Oncology, and Tumor Immunology (Campus Charité Mitte), Medical Department, Charité University Medicine Berlin, 10117 Berlin, Germany; 6 Anji Pharmaceuticals, Cambridge, MA 02142, USA

**Keywords:** fibroblast growth factor receptor, amplification, mutation, fusion, FGFR inhibitor, predictive biomarker, clinical trials

## Abstract

Inhibitors of fibroblast growth factor receptor (FGFR) signaling have been investigated in various human cancer diseases. Recently, the first compounds received FDA approval in biomarker-selected patient populations. Different approaches and technologies have been applied in clinical trials, ranging from protein (immunohistochemistry) to mRNA expression (e.g., RNA in situ hybridization) and to detection of various DNA alterations (e.g., copy number variations, mutations, gene fusions). We review, here, the advantages and limitations of the different technologies and discuss the importance of tissue and disease context in identifying the best predictive biomarker for FGFR targeting therapies.

## 1. Introduction

Growth factor signaling has been identified as a major hallmark of cancer, leading to dysregulation of survival, growth and metabolic pathways [1]. Aberrant growth factor signaling via, e.g., mutations in tyrosine kinase domains, or overexpression or amplification of cognate receptors has recently also been linked to modulation of tumor microenvironment and, thus, plays a major role in controlling immune response against cancers [2,3].

Consequently, these pathways were identified early on as potential targets for cancer therapy by either inhibiting downstream signaling (e.g., alpelisib or copanlisib targeting phosphoinositide-3-kinase (PI3K), temsirolimus and other rapalogs targeting mammalian/mechanistic target of rapamycin (mTOR) or trametinib targeting mitogen-activated protein kinase kinase (MAPK/MEK)) or by directly interfering with upstream receptors. Seminal insights into growth factor receptor blockade were obtained from cetuximab and panitumumab targeting epithelial growth factor receptor (EGFR), trastuzumab targeting human epidermal growth factor receptor 2 (Her2/neu, erb-B2) and bevacizumab targeting vascular endothelial growth factor (VEGF). For the first two targets, a clear predictive biomarker selection technique was developed, ranging from simple target expression to downstream mutational profiles [4,5]. However, no such strategy is currently available for anti-angiogenic agents [6].

Recently, also, the fibroblast growth factor receptor (FGFR) family has entered the focus of drug development and the first compounds, erdafitinib (JNJ-42756493, Balversa™), pemigatinib (INCB054828, Pemazyre™), infigratinib (BGJ398, Truseltiq™) and, most recently, futibatinib (TAS-120, Lytgobi™), have received United States Food and Drug Administration (US FDA) approval for treatment of urothelial (bladder) cancer and intrahepatic biliary tract cancer, respectively [7,8]. While these US FDA-approved compounds use FGFR gene fusions and mutations as a companion diagnostic for patient selection, other agents use FGFR gene amplifications or FGFR overexpression to select patients in their respective clinical trials [9,10,11]. In this article, we will summarize and review the different approaches used for identification of patients for small-molecule FGFR inhibitor clinical trials in various cancer indications.

## 2. Brief Introduction to FGFR Biology and Signaling

**Figure 1:**
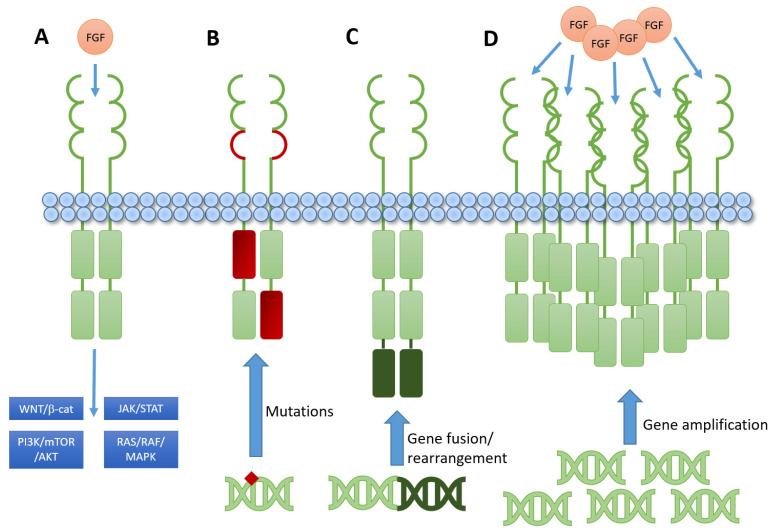
Schematic representation of FGFR signaling and impact of various alterations. (**A**) Physiologic signaling upon ligand binding leads to various downstream signaling cascades affecting cellular survival, growth, migration, metabolism and interaction with cellular microenvironment. (**B**) Point mutations (marked in red) lead to constitutive activation by either affecting the extracellular ligand-binding domain or the intracellular tyrosine kinase domains. Signaling becomes independent of FGF ligand binding. (**C**) Gene fusions, rearrangements or translocations on DNA level (marked in dark green) lead to ligand-independent constitutive activation of the kinase domains by adding alternative kinase elements. (**D**) Gene amplification by DNA copy number alterations leads to higher expression of the receptor, providing more opportunities for ligands to bind and to activate the signaling cascade. It is noteworthy that all shown alterations also lead to increased mRNA expression levels but not all alterations lead to receptor overexpression. AKT: synonymous Protein Kinase B; β-cat: β-catenin; FGFR: fibroblast growth factor receptor; JAK: janus kinase; MAPK: mitogen-activated protein kinase; mTOR: mammalian/mechanistic target of rapamycin; PI3K: phosphoinositide-3-kiase; RAF: rapidly accelerated fibrosarcoma; RAS: rat sarcoma; STAT: signal transducer and activator of transcription; WNT: wingless and Int-1.

Five fibroblast growth factor receptors (FGFRs) have been identified in humans, with four of them belonging to the family of transmembrane receptor tyrosine kinases and contain three immunoglobulin-like extracellular domains, mediating ligand specificity, a transmembrane spanning domain and an intracellular tyrosine kinase domain [12,13,14,15]. Twenty-two ligands for FGFRs have so far been identified in humans. They are usually subclustered into intracrine, endocrine and paracrine members. Except for intracrine fibroblast growth factor ligands (FGFs; FGF11-FGF14) that signal via the canonical receptor pathways, each FGF can bind to multiple FGFRs, leading to a complex interaction map of ligands and receptors (for more detail, see our previous review [16]). Ligand binding leads to receptor dimerization and subsequent phosphorylation of downstream signaling molecules (mostly fibroblast growth factor receptors substrate 2α, FRS2α, and growth factor receptor-bound protein 2, GRB2) into the PI3K-AKT or the RAS-RAF-MAPK pathway, which regulate cellular survival and growth mechanisms [17,18,19]. Other downstream targets include signal transducer and activator of transcription (STAT) molecules [20], adhesion molecules, such as N-cadherin [21,22,23] or the WNT/β-catenin pathway, thus, linking FGFR to invasion and metastasis formation [24,25,26] as well as chemoresistance via epithelial-mesenchymal transition [27,28] (Figure 1). Unlike FGFR1-3, FGFR4 does not have splice variants in IgIII, which generates the IIIb and IIIc transcript variants encoding different receptor isoforms. The lack of alternative splicing of IgIII reduces the ability to switch ligand binding specificity [29,30]. FGFR4 contains a unique amino acid (Cystein at position 522, Cys522) in the kinase domain, which is not present in FGFR1–3. Cys552 is conserved in just five other human protein kinases, including MK2, MK3, S6K2, STK40 and TTK. Thus, covalently targeting the Cys552 in FGFR4 is an appealing strategy for achieving selective inhibition of FGFR4, both with respect to isoform and kinome selectivity. This structural difference enables the design of FGFR4-specific inhibitors [31]. Selective inhibitors of FGFR4 have demonstrated clinical benefit in HCC patients with high FGF19 expression [32].

Overall, genomic alterations in FGFRs have been identified in approximately 6–7% of all human cancers [33,34,35]. The oncogenic potential of FGFRs has been linked to increased expression due to gene amplification (up to 66%), gene fusion/translocation (up to 8%) or gene mutations (up to 26%) in the signaling domains or abnormity of FGFR ligands [36,37,38]. It is interesting that these alterations show a distinct prevalence pattern in various human tumor types. While the frequency of FGFR2 mutations is highest in endometrial cancer (10–12%), it is below 5% in, e.g., non-small-cell lung cancer (NSCLC), gastric and urothelial cancer. Yet, FGFR3 mutations reach a prevalence of 75% in non-muscle-invasive bladder cancer, while it drops to 15% in the more aggressive muscle-invasive subtype [34,39,40,41]. Similar findings were observed for amplifications, which are rare for FGFR3 and FGFR4, while FGFR1 amplification was found in up to 19% of NSCLC and hormone-receptor-positive breast cancer (dropping to 4% in triple-negative breast cancer) [42,43,44,45]. Amplification of FGFR2 was found in up to 10% of gastric cancers [46,47] and FGFR2 gene fusions in up to 20% of intrahepatic cholangiocarcinomas and up to 6% of urothelial carcinomas [35]. Most prominent FGFR gene fusions represent FGFR3-TACC3 (transforming acidic coiled-coil containing protein 3) in urothelial cancer and FGFR2-BICC1 (BicC family binding protein 1) or FGFR2-AHCYL1 (adenosylhomocysteinase like 1) in intrahepatic cholangiocarcinomas [48,49,50,51]. These results indicate that expression of and genetic alterations in the various FGFR isoforms are highly context dependent and that a thorough understanding of the (dys-)regulated FGFR pathway is important to understand the optimal therapeutic setting for FGFR inhibitors. Recent data also suggest epigenetic mechanisms, such as methylation or miRNA expression, to regulate FGFR expression per se or in response to treatment, thus, representing a resistance mechanism [52,53,54].

It was demonstrated that FGFR2 is able to induce expression of programmed cell death 1 ligand 1 (PD-L1) via the janus kinase (JAK)/STAT pathway in colorectal cancer [55] and a non-T-cell-inflamed phenotype was observed in FGFR3-driven urothelial cancer [56,57], although a recent study from Denmark could not confirm this finding [58]. Yet, preclinical data clearly demonstrate that inhibition of FGFR enhances the infiltration of CD8+ T cells and inhibits tumor growth via modulation of the tumor microenvironment [59,60,61]. Wu et al. observed that the FGFR inhibitor-mediated blockade of the mitogen-activated protein kinase (MAPK)/extracellular-signal-regulated kinase (ERK) pathway in cancer-associated fibroblasts leads to diminished proliferation, migration and secretion of the vascular adhesion molecule 1 (VCAM-1) in these cells, which promotes T cell infiltration by breaking down the tumor/stroma barrier [62]. Furthermore, FGFR tyrosine kinase inhibitors were able to upregulate major histocompatibility complex (MHC) class I and class II expression via induction of the MHC Class II gene master regulator Class II transactivator (CIITA) and subsequent inhibition of MAPK and to augment the antitumor effects of FGFR1-reactive T cells [63]. Overall, the non-canonical effects of FGFR inhibitors in regulating the immune phenotype of tumors is still poorly understood and needs more experimental and clinical studies.

For more details on the metabolic pathways affected by FGFR signaling in the liver, we refer to other recently published reviews [64,65].

## 3. FGFR Inhibitors in Clinical Trials

This article focuses on small-molecule inhibitors of FGFR signaling, although other modalities, such as FGF ligand traps (e.g., GSK3052230), FGFR2-targeting antibody drug conjugates (e.g., aprutumab ixadotin/BAY 1187982) or receptor-blocking antibodies (e.g., bemarituzumab), have also been explored in early clinical trials [41].

Most of the small-molecule FGFR inhibitors (Table 1) are ATP-competitive inhibitors of several FGFR isoforms. Commonly, FGFR1–3 are inhibited at low nanomolar concentrations in a biochemical assay, while inhibition of FGFR4 is often less potent. Selective inhibitors of FGFR4, such as fisogatinib, roblitinib or H3B-6527, are irreversible covalent inhibitors, as is futibatinib that represents the only irreversible pan-FGFR inhibitor. Allosteric inhibitors, such as Alofanib (RPT835) or SSR128129E, have not yet reported human clinical data [66,67]. For many of the compounds listed in Table 1, clinical trials are still ongoing (please see [41], also for details on response rates and www.clinicaltrials.gov for more information, last accessed on 3 October 2022). So far, only erdafitinib, infigratinib, pemigatinib and futibatinib have received FDA approval for treatment of bladder cancer or intrahepatic cholangiocarcinoma.

The majority of the FGFR inhibitors that were used in clinical trials represent pan-FGFR inhibitors that usually target FGFR1, FGFR2 and FGFR3 at low-nanomolar IC50 values and FGFR4 at slightly higher values. Most of the compounds used a non-biomarker-selected all-comer population for dose escalation and switched to a distinct patient population for dose expansion and later phases of development. The tumor-agnostic potential of FGFR inhibitors, regardless of the underlying FGFR subtype altered, was recently demonstrated in the RAGNAR clinical trial with erdafitinib [68], in NCI-MATCH trial EAY131 with AZD4547 and in a large phase 1 study with rogaratinib [9]. Taken together, responses were observed in over 25 different malignancies. Interestingly, only the FGFR4-selective inhibitors explore the ligand FGF19 as a potential patient selection biomarker, while all other compounds focus on FGFR alterations, such as gene fusions, amplifications or mutations. As outlined above, these alterations show differential patterns in various human tumor types. Thus, assays for prospective patient selection either need a broad specificity or multiple assays need to be applied.

In the following section, different assays used in clinical development or as a full companion diagnostics tools for FGFR inhibitors will be discussed.

## 4. Predictive Biomarkers for FGFR Inhibitors

Early clinical trials with small-molecule FGFR inhibitors focused solely on histological tumor entities with a reasonable frequency of FGFR DNA alterations. FGFR1 amplification is the most frequently observed DNA abnormality in the squamous subtype of NSCLC (up to 20%). Meanwhile, it became obvious that not all NSCLC patients with an FGFR copy number gain had higher FGFR expression levels [104]. In gastric cancer, it could be shown that only homogenous FGFR2 gene amplification led to FGFR2 overexpression and, thus, to a treatment benefit [105]. A weak overlap between FGFR1 amplification and FGFR1 overexpression was also described in a large cohort of head and neck squamous cell carcinoma (HNSCC) patients [106].

Given the mode of action of small-molecule FGFR kinase inhibitors, the enzymatic activity can only be inhibited if a) there is a higher FGFR expression level within the tumor or b) if the enzymatic activity is increased by an activating single-nucleotide variant within the kinase domain. Very interestingly, in urothelial carcinoma, where FGFR3-activating mutations are most frequent (see before), it could be shown that the presence of a point mutation leads to strong overexpression of the mutant protein. Similar findings were observed for FGFR3 translocations in urothelial carcinoma and for FGFR2 fusions in cholangiocarcinoma, where the lack of the 3′ end of the FGFR transcript being fused to another partner gene delays the micoRNA-mediated degradation of the fusion transcript and, thus, increases FGFR fusion gene tumor expression levels accordingly. Thus, the selection of patients eligible for FGFR inhibitor therapy can be referred back to the degree of the FGFR overexpression within the tumor, surprisingly, even including overexpression of FGFR due to activating point mutations. For urothelial cancer, it has been known for decades that some tumors, especially in earlier stages of the disease, reveal FGFR overexpression without an underlying FGFR DNA alteration [107,108,109], e.g., an activating FGFR3 mutation or an FGFR3 gene fusion. It remained a matter of speculation if patients without such a DNA alteration may also benefit from FGFR inhibitor therapy. Schuler et al. demonstrated FGFR inhibitor sensitivity in FGFR-overexpressing urothelial carcinoma patients in the absence of a detectable FGFR DNA alteration [9]. A recent Phase 2 study in gastric cancer with the FGFR2-targeting monoclonal antibody bemaritizumab demonstrated FGFR2 overexpression via immunohistochemistry in 30% of screened patients, while other datasets suggest that the amplification of FGFR2 in tissue reaches 2.2–4% and 7.7% via ctDNA analysis [110,111]. Patients with overexpression of FGFR2b, even without ctDNA amplification, demonstrated a benefit from the addition of bemarituzumab to mFOLFOX6, supporting further evaluation of bemarituzumab in tumors with FGFR2b overexpression, regardless of an underlying FGFR2 gene amplification.

Recent data from the indication-agnostic NCI-MATCH Trial EAY131 (subprotocol W) also confirmed that alterations in FGFR, as detected by next-generation sequencing (NGS), in that tumor tissue is more common across a broad range of tumor diseases than previously expected. Here, various alterations were found also in, e.g., salivary gland tumors, rectal cancer, pancreatic cancer, prostate cancer and other tumors that were considered not to harbor FGFR alterations [74]. These findings corroborate the results from Schuler et al., who could demonstrate a higher proportion of FGFR-overexpressing patients in their study than expected from The Cancer Genome Atlas (TCGA) data [9], e.g., 57% vs. 13% for HNSCC or 46% vs. 30% for squamous NSCLC, whereas data for gastric cancer (19% vs. 18%) or lung adenocarcinoma (11% vs. 12%) matched the database prediction. Interestingly and unexpectedly, it could also be demonstrated in this study that unusual alterations could be detected in the cohort of urothelial cancer patients. Here, 5.5% of enrolled patients showed overexpression of FGFR1 and several cases showed double positivity for FGFR1/2, FGFR1/3 or FGFR2/3 overexpression, which would not be detected by a FISH approach [112]. Still, a direct comparison of the prevalence of FGFR pathway alterations between different studies or databases needs to be conducted with some precautions, since different cut-offs or methods were applied that may lead to different results.

**Figure 2:**
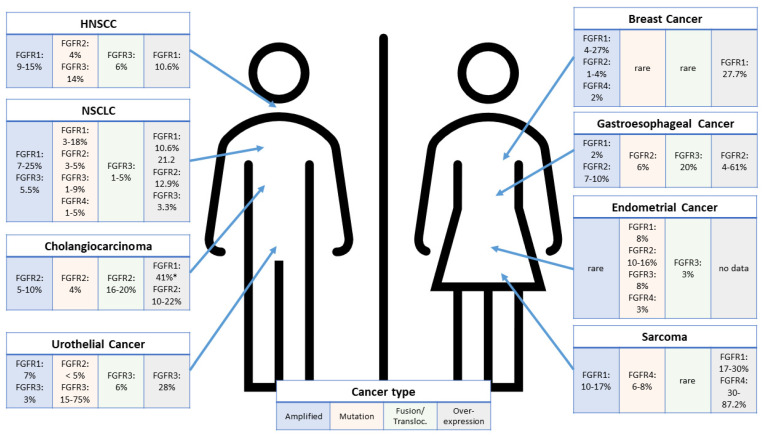
Prevalence of FGFR alterations in selected tumor types. For each tumor type, the prevalence of amplifications, mutations, fusions or translocations and overexpression is highlighted according to [34,35,36,37,38,39,40,42,43,44,45,46,47,48,49,50,51,113,114,115,116,117,118,119,120,121,122,123,124,125,126]. Overexpression relates to protein overexpression as (usually) detected via immunohistochemistry. FGFR1 data marked with * for cholangiocarcinoma represent mRNA expression data. The most prevalent alteration is depicted in bold for each tumor type. FGFR: fibroblast growth factor receptor; HNSCC: Head and Neck Squamous Cell Carcinoma; NSCLC: Non-Small-Cell Lung Cancer.

Taken together, these findings point to the direction that cancer patients with FGFR tumor overexpression, even in the absence of an underlying FGFR DNA alteration, could benefit from FGFR inhibitor therapy (Figure 2 & Table 2). As receptor tyrosine kinases, including FGFRs, are challenging targets for immunohistochemistry (IHC)-suited antibody generation, we discuss, in the following chapter, alternative ways to quantify FGFR expression using archival tumor biopsy specimens in order to identify patients most likely to benefit from FGFR inhibitor therapy.

### 4.1. Immunohistochemistry

FGFR inhibitors currently available for clinical use are usually small-molecule inhibitors of the tyrosine kinase function of FGFRs. Therefore, directly detecting the drug target by immunohistochemistry was considered to be the best predictive and patient selection biomarker for these compounds and the technology would be readily available for decentralized testing in local pathology labs. Except for FGFR2-specific antibodies or FGFR2-targeting antibody drug conjugates, none of the small-molecule inhibitors listed in Table 1 currently use IHC as a predictive biomarker. The FGFR4-specific inhibitor Fisogatinib uses IHC to detect the FGFR ligand FGF19 [32], although recently, mRNA analysis was also used in this setting [127].

The use of immunohistochemistry for small-molecule FGFR inhibitors is further limited by the fact that these drugs are usually pan-FGFR inhibitors and, currently, there is no antibody available that detects all necessary isoforms simultaneously and with the needed sensitivity and specificity. Therefore, multiplex approaches using several isoform-specific antibodies would be needed, which are technically challenging to develop due to limited separability of chromogenic substrates. Fluorescence labels might overcome this technical limitation but would require specific technologies in the analysis labs, which limits the market access for this approach.

Overall, immunohistochemistry is not recommended as a predictive or patient selection biomarker for FGFR inhibitors.

### 4.2. FISH/CISH to Detect Gene Copy Number Variations

Several FGFR inhibitors have used fluorescence in situ hybridization (FISH) or chromogenic in situ hybridization (CISH) to detect gene copy number variations (CNV), since retrospective data showed a good correlation between high protein expression, as detected by immunohistochemistry, and CNV, e.g., for FGFR2 in gastric cancer [116], where a copy number gain is also associated with lymphatic invasion and poor prognosis [128]. Interestingly, the reverse correlation between CNV and protein expression could not be confirmed for FGFR1 in NSCLC patients, although FGFR1 amplification was also associated with poorer overall and disease-free survival here [129]. It is now confirmed that only high CNVs actually translate into high protein expression, which decreases the prevalence of FGFR-positive patients, usually to a low percentage of the overall population (e.g., 4% for FGFR2-amplified gastric cancer [130,131]). This raises the question for the right cut-off to achieve clinical efficacy, since only highly amplified cancers seem to be dependent on FGFR signaling and show higher sensitivity towards small-molecule inhibitors, such as AZD4547 [132]. Furthermore, there is great intratumor heterogeneity in FGFR CNVs and amplifications are not evenly distributed, which leads to the development of complex evaluation scores. In lung cancer, Schildhaus et al. proposed that high-level amplification is defined as FGFR1/centromere 8 (CEN8) ratio ≥ 2.0, or average number of FGFR1 signals per tumor cell nucleus ≥ 6 or the percentage of tumor cells containing ≥ 15 FGFR1 signals or large clusters ≥ 10% [133]. Such scores require extensive training of the pathologist to minimize interobserver variability, since selecting different scoring criteria may lead to different results. Interestingly, although FGF19 CNV could be detected by FISH and has been shown to correlate with response to the multi-kinase inhibitor sorafenib in HCC [134], current FGFR inhibitor trials either employ mRNA expression or protein overexpression via immunohistochemistry for FGF19 [32]. Similar to fluorescence-labeled immunohistochemistry, FISH requires specialized technical equipment that limits the broad application of this assay. CISH, which could overcome these limitations, is currently not used in any clinical trial.

### 4.3. mRNA Expression Technologies

RNA in situ hybridization (RNA-ISH) was considered to be challenging in clinical trial settings. However, the introduction of the RNAscope technology in 2012 allows for sensitive and specific detection of individual mRNA molecules, also in paraffin-embedded tissue specimens, due to a novel probe design. It preserves spatial resolution and tissue architecture and can be used with chromogenic or fluorescent detection systems and is, therefore, highly multiplexable and results are well quantifiable [135].

The Nanostring nCounter technology was first introduced in 2008. It is a highly multiplexable direct measurement of mRNA expression with high sensitivity and reproducibility and low detection limit, also from paraffin-embedded tissues [136]. In contrast to mRNA in situ hybridization, but similar to PCR or sequencing technologies, it does not maintain the spatial resolution of signals, since a direct digital readout is performed. However, new algorithms allow one to recalculate stroma/tumor content and, thus, provide additional information on signal distribution.

Preclinical data indicated that, actually, mRNA expression is a better predictor for FGFR sensitivity than CNV or other biomarkers [103,106]. Schuler et al. were the first to adopt those technologies for prospective selection of patients in a Phase 1 study in an indication-agnostic manner [9]. In addition to demonstrating the technical feasibility and robustness in a global multi-center trial, they could demonstrate that mRNA technologies identified a much broader patient population (including patients without apparent genetic alteration) than anticipated, thus, broadening the potential to bring benefit to patients. These data were recently confirmed for FGFR1–4 mRNA expression in breast cancer [137]. While Nanostring technology is highly sensitive and multiplexable, it is also considered expensive and requires bioinformatics workup to calculate tumor content. RNAscope offers the advantage of maintaining the spatial resolution, which may provide useful additional information on distribution of signals. It is considered fast and provides an IHC-like readout but does require intensive training of the pathologist or a central reading in clinical studies to minimize interobserver variability

In addition, several PCR-based technologies (e.g., digital droplet PCR or quantitative reverse transcriptase PCR) have been developed and used in clinical trials, usually only on a retrospective basis and not for patient selection. This is also the case for RNA sequencing.

### 4.4. Next-Generation DNA Sequencing (NGS)

Massive parallel sequencing, now usually called next-generation sequencing (NGS), was developed in the mid-1990s and allows for the rapid reading of DNA fragments up to 400 base pairs in length and a maximum readout of up to 1 terabase per run. This provides information on multiple genes or genetic aberrations, including mutations, fusions and copy number variations, which is of special interest for FGFR inhibitors. First kits, e.g., Foundation One, have received FDA approval as a CDx test for various drugs and indications. Currently, pemigatinib and infigratinib use Foundation One as an FDA-approved companion diagnostics test (CDx) for FGFR2 fusions in intrahepatic cholangiocellular carcinoma [138].

In general, NGS approaches are considered the gold standard for molecular testing in cancer. Their availability and reimbursement are constantly growing, since they allow one to obtain information on numerous druggable genetic alterations at once, thus, limiting the need for tissue and specific sequencing requests [139]. NGS results are complex and contain information in several genetic alterations, which requires expert discussion in molecular tumor boards, since, currently, no hierarchy of results is established, which would guide treatment decisions in the case of multiple druggable hits. The benefit of obtaining multiple insights at once is, of course, that guidance on resistance mechanisms and on sequencing of therapeutic approaches could be discussed up front.

A limitation of NGS is its rather long turnaround time of up to 4 weeks and the comparably high costs of the approach. The latter one is a major hurdle when using NGS to identify rare genetic alterations with low prevalence in a certain population, which often makes the use of NGS prohibitive, even in clinical trials.

### 4.5. NGS of ctDNA (Liquid Biopsies)

To overcome limitations related to tissue-based testing, such as invasiveness, biopsy sampling error, intra-tumor heterogeneity or scarcity of available tissues, liquid biopsies have been included in various clinical trials. Here, circulating free tumor DNA (ctDNA) can be analyzed by means of next-generation sequencing and alterations, meaning mutations or fusions can easily be detected. Furthermore, the level of ctDNA itself has been shown to be an early prognostic marker correlated to disease recurrence in, e.g., NSCLC or colorectal cancer [140,141,142].

Jogo et al. demonstrated that ctDNA analysis detects FGFR2 amplification in gastric cancer at a higher frequency than tissue analysis (7.7% vs. 2.6–4.4%). They could also identify patients where FGFR2 amplification was detectable only in liquid biopsy but not in a paired tissue sample and that these cases had an overall worse prognosis [110]. Further data are needed to validate if only high-level amplifications (CNV > 5) translate into ctDNA positivity or if also lower-copy-number changes could be detected in liquid biopsies [132].

An important application of NGS analyses to ctDNA samples is the monitoring of resistance development. It was demonstrated that FGFR inhibitor treatment can lead to secondary mutations in FGFR2 cholangiocarcinomas with FGFR2 alterations, which drives resistance. Here, ctDNA analyses were applied longitudinally and could overcome the observed intratumor heterogeneity and polyclonality in assessing resistance mutations [143,144]. This approach was also used to inform treatment decisions in patients who developed resistance against infigratinib or Debio 1347 and who could still benefit from subsequent treatment with the irreversible FGFR inhibitor futibatinib [145]. Interestingly, resistance to the CDK4/6 inhibitor ribociclib, in combination with fulvestrant in ER+ breast cancer, can be mediated by amplification of FGFR1. ctDNA analysis from the registrational MONALEESA-2 trial confirmed that patients with FGFR1 amplification had shorter progression-free survival than wild-type patients [146].

Overall, these data demonstrate the high diagnostic, prognostic and predictive value of NGS-based detection of FGFR pathway alterations. Specifically, the sophisticated longitudinal monitoring of resistance development has great potential to improve treatment strategies due to advanced and adaptive therapeutic schedules for patients.

## 5. Discussion

The low concordance between FGFR amplification and FGFR overexpression, as described for large datasets from NSCLC [104] and HNSCC [106] patients, questions to what extent the pre-selection of patients based on FGFR amplifications may have led to the failure of early clinical trials with FGFR inhibitors [147]. In addition, even for the very same FGFR alteration within the same tumor type, highly different prevalence data are reported across published data: a recent meta-analysis on the frequency of FGFR1 gene amplification in NSCLC evaluating twenty-three studies (5252 patients) revealed a 10-fold difference in the prevalence, ranging from only 4.9 to up to 49% [148]. This high variability raises the question if an additional layer of complexity to define an FGFR-positive patient is based on the method used to detect the FGFR alteration, which all have a different sensitivity and/or specificity. In addition, the definition of FGFR positivity might be improved by applying, in parallel, two different methods to detect FGFR alterations or to perform even orthogonal assays, e.g., evaluating FGFR DNA alterations and FGFR expression levels using the same tumor tissue biopsy specimen. To date, only limited data on FGFR positivity, confirmed by two different readouts, are available for patients treated with an FGFR inhibitor. However, recent data from the Phase 2/3 study FORT-1, evaluating rogaratinib in first-line urothelial cancer, revealed a very high response rate of 52.4% in a small subgroup of patients pre-selected for FGFR1/3 mRNA overexpression and retrospectively confirmed of having, in addition, either an underlying FGFR3-activating mutation or an FGFR3 gene fusion compared to the ORR of 19.5% in patients being positive for FGFR1/3 mRNA overexpression, regardless of whether an underlying FGFR DNA alteration was detected [149]. This points to a higher benefit from FGFR inhibitor therapy in patients with FGFR positivity, confirmed by two different readouts and, ultimately, leads to the provocative question of whether the therapeutic impact of existing FGFR inhibitors is rather limited by the difficulties to define an FGFR-positive tumor than by the drugs themselves.

**Figure 3:**
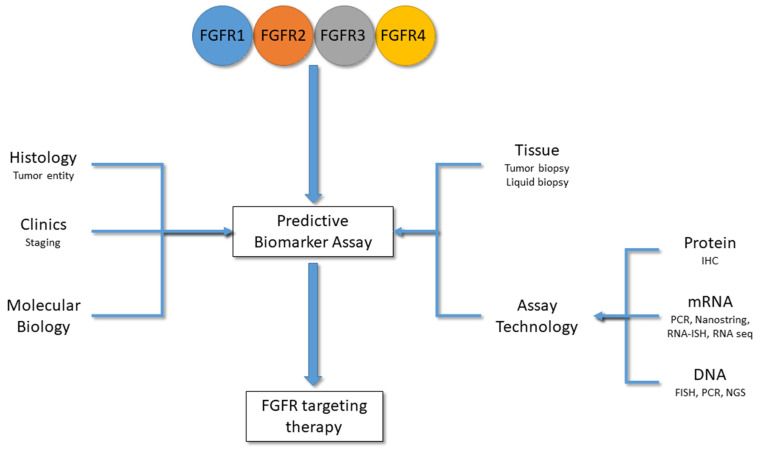
Factors influencing the selection of a predictive biomarker assay for FGFR-inhibitor therapies. Alterations in FGFR1-4 impact on the predictivity of a biomarker assay. In addition to the molecular biology of the alterations (CNV, fusion, mutation, etc.), also, the underlying tumor entity (histology), the clinical staging (e.g., muscle-invasive vs. non-muscle-invasive bladder cancer), tissue availability and the assay technology with different target readouts (protein, mRNA, DNA) determine which FGFR targeting therapy would bring benefit to a patient.

In addition to technical reasons for choosing a certain predictive biomarker assay, also, clinical and molecular factors need to be taken into consideration to find the best therapy for patients (Figure 3). Still, several key questions remain elusive when selecting a predictive FGFR biomarker assay.

First, gene fusions are considered strong oncogenic drivers and recent clinical data show strong efficacy of compounds targeting, e.g., neuregulin 1 (NRG1) [150,151,152], neurotrophic tyrosine kinase receptor (NTRK) [152,153] or anaplastic lymphoma kinase (ALK) [154,155] fusions in patients across various tumor types. Despite selecting patients for FGFR gene fusions, clinical responses to FGFR inhibitors seem less deep and less pronounced than compared to other fusion-specific agents, such as Larotrectinib. This may be due to the complex crosstalk and redundancy within the FGFR signaling network, which leads to a basic physiologic signaling to maintain metabolic and tissue homeostasis functions [16,64]. For example, whilst the normal, non-fusion-bearing NTRK protein does not play any role outside the central nervous system in adults and the physiological roles of ALK1 proteins in adults are still a matter of debate, FGFR proteins exert many physiological functions. Thus, the fact that non-malignantly transformed somatic cells express baseline FGFRs (e.g., FGFR3 in normal urothelial cells) separates FGFR proteins clearly from NTRK proteins, where adult somatic cells lack expression and, thus, downstream signaling. In addition, the continuous physiological FGFR background signaling active in normal body cells [16,64] could also explain the early and rapid development of resistance bypass pathways under FGFR inhibitor treatment, as demonstrated for, e.g., the upregulation of ErbB family members after infigratinib treatment or for upregulation of the EGFR pathway [156,157]. A common downstream mediator of receptor tyrosine kinases is the PI3K/AKT/MAPK pathway. Alterations in this pathway, downstream of the receptor, have also been described to confer resistance to FGFR inhibitors [158,159] and could, in turn, be overcome again by combination therapy with an MEK inhibitor [160]. Insights into the parallel occurrence of such resistance mutations could, therefore, improve the response rates of FGFR inhibitors and favor the use of NGS panel approaches to select patients for FGFRi treatment. Interestingly, also for the approved FGFR inhibitors, differences in efficacy were observed based on the underlying FGFR DNA alteration. For pemigatinib, all observed responses were limited to FGFR2 fusion-positive cholangiocellular carcinoma and no confirmed responses were seen in other FGFR alterations [98]. In urothelial cancer, in contrast, long-term follow-up of a phase 2 study of erdafitinib revealed that duration of response and overall survival were generally similar between patients with FGFR mutations and those with FGFR fusions [161], which renders clinical decision-making challenging. In comparison to other growth factor receptor pathway inhibitors, e.g., Osimertinib against EGFR, agents targeting FGFRs seem have a lower overall potency, which indicates that tumor cells may be less addicted to complex FGFR signaling pathways with their multiple redundancies.

Second, it is intriguing that the same FGFR DNA alteration (mutation, fusion, amplification) leads to different sensitivity to FGFR inhibitors, depending on the underlying histologic subtype. Across several compounds, FGFR2 fusions were most sensitive in intrahepatic cholangiocellular carcinoma (ihCC) and FGFR3 mutations showed the best responses in urothelial cancers. FGFR2 amplification gave positive results in gastric cancer but disappointed in, e.g., breast cancer or NSCLC. It is unclear why different tumor types show different dependencies on these FGFR pathway alterations, even when DNA-independent biomarkers, such as mRNA overexpression, are applied. So far, only limited data are available on potential co-mutations or further downstream alterations, but it is obvious that tumor type and histology matter [74].

Third, the overall prognostic and predictive value of (different) FGFR alterations remains unclear. It is also unclear what a biologically meaningful cut-off for the different assay formats discussed above would be and a clear threshold for FGFR positivity is currently not available. As an example, approved CDx tests for FGFR inhibitor treatment, based on FGFR mutations or fusions, apply a cut-off for FGFR positivity as a mutant allele fraction (MAF) of at least 5%, whereas an NGS-based mutation test may easily detect an MAF of 0.1% or even less. However, in contrast to the established cut-off for the MAF for EGFR-activating mutations shown to be clinically meaningful for EGFR inhibitor treatment [162], no such correlation of MAF and clinical response has been shown for any FGFR inhibitor to date. The same applies for the correlation between the degree of FGFR protein or FGFR mRNA overexpression and clinical response when being used for patient selection in clinical trials or for the cut-off definition of a clinically meaningful copy number gain.

Only limited data are available that confirm a negative prognosis for urothelial cancer patients with FGFR genomic alterations [163], while other data could not establish a correlation to overall survival (OS) or progression-free survival (PFS) or response to systemic therapy [164]. Several studies recently investigated the combination of FGFR inhibitors with immune checkpoint inhibitors. While a recent study from Denmark could not identify a statistically significant correlation between FGFR3 amplifications or mutations to PD-L1 expression in primary urothelial carcinomas [58], Sweis et al. showed that an activated FGFR3 pathway is linked to non-T-cell-inflamed tumors, which are characterized by poorer prognosis and resistance to immune checkpoint inhibitors [56]. This may be due to the activation of neural-precursor-cell-expressed developmentally down-regulated protein 4 (NEDD4), an E3 ubiquitin ligase, by activated FGFR3 that could lead to proteasomal degradation of PD-L1, indicating the potential of combination therapy in this setting [165]. FGFR3 mutations, therefore, seem to be a negative predictor to immunotherapy response in urothelial cancer. However, promising data from erdafitinib and rogaratinib combination trials with PD-1 antibodies indicate that parallel inhibition of overactive FGFR signaling in urothelial cancer may be a pre-requisite to sensitize tumors to the benefit of subsequent checkpoint inhibitor therapy [166,167].

## 6. Conclusions

Drugs targeting the FGFR pathway have matured and received approval for various cancer indications, which require prospective biomarker testing. Several technologies, ranging from immunohistochemistry to PCR or sequencing technologies and to gene expression approaches, have been investigated in clinical trials and provide different advantages and limitations. Next-generation sequencing is approved as a companion diagnostic kit for two compounds but further understanding of the role of distinct alterations (e.g., gene fusions vs. mutations vs. overexpression) is urgently needed, as a one-fits-all approach does not seem successful for FGFR inhibitors. The available clinical data indicate that the nature of the alteration and the underlying cancer disease itself significantly impact the predictivity of those biomarkers and more research is needed to obtain clarity for clinicians and patients on what biomarker cut-off and what test achieve the best results in a certain disease context.

Regarding the knowledge of advantages and limitations in FGRR tests, the following recommendations can be given: In situ hybridization approaches allow one to determine the gene copy number, which seems to be a less reliable predictor of treatment outcome. Immunohistochemistry is currently not recommended as a screening method in view of the possibility of NGS assays. Depending on the available patient sample (including the possibility to achieve tumor content enrichment through tissue microdissection or the alternative use of liquid biopsies), molecular pathological testing of potential FGFR mutations or fusions should be performed using DNA/RNA-based NGS platforms.

## Figures and Tables

**Table 1:** Clinically tested FGFR inhibitors.

Name	Selectivity	Indications	Phase	Biomarker	Reference
ASP5878	FGFR1-4	UC, HCC, sqLC	1	FGFR3 fusion or mutation by FISH or PCR (UC), FGF19 overexp (HCC) or FGFR1 overexp (sqLC) by IHC	[69,70]
AZD4547	FGFR1-4	BC, GC, sqLC, agnostic	3	FGFR copy number in ctDNA (BC), FISH (GC, sqLC), any FGFR alteration by NGS in the indication agnostic setting	[11,71,72,73,74]
Debio 1347	FGFR1-3	Advanced solid tumors	1/2	FISH, NGS	[75,76]
Derazantinib (ARQ087)	FGFR1-3	ihCC	1/2	FGFR2 fusion by FISH or NGS	[77,78]
Dovitinib (TKI-258)	FGFR1 & 3	RCC and other solid tumors	3	No specific biomarker used	[79,80]
E7090	FGFR1-3	GC, ihCC, advanced solid tumors	1/2	FGFR2 amp (GC), FGFR2 fusion (ihCC), NGS	[81,82,83]
Erdafitinib (JNJ-42756493)	FGFR1-4	UC	approved	FGFR2/3 alterations by qRT-PCR	[84,85]
Fisogatinib (BLU-554)	FGFR4	HCC	1/2	FGF19 by IHC	[32]
Futibatinib (TAS-120)	FGFR1-4	ihCC, GC, advanced solid tumors	approved	FGFR2 amp (GC), various FGFR aberrations	[86,87,88,89]
Infigratinib (BGJ398)	FGFR1-3	ihCC, gliomas	approved	Any alteration of FGFR1 or FGFR3 (gliomas) or FGFR2 (ihCC)	[90,91,92]
LY2874455	FGFR1-4	GC, NSCLC	1	FGFR1 amp (NSCLC), FGFR2 amp (GC)	[93,94]
ODM-203	FGFR1-4	Advanced solid tumors	1	Any genetic FGFR aberration	[95,96]
Pemigatinib (INCB054828)	FGFR1-3	ihCC	approved	NGS	[97,98]
Ponatinib	FGFR1-4	ihCC	3	FGFR2 fusion/rearrangement by FISH or NGS	[99,100]
Roblitinib (FGF401)	FGFR4	HCC	1/2	FGFR4 expression by PCR	[101]
Rogaratinib (BAY 1163877)	FGFR1-4	Advanced solid tumors	1/2	mRNA expression (RNA-ISH, Nanostring)	[9,102,103]

Amp: amplification; BC: breast cancer; FISH: fluorescence in sitru hybridization; GC: gastric cancer; HCC: hepatocellular carcinoma; ihCC: intrahepatic cholangiocarcinoma; IHC: immunohistochemistry; NGS: next generation sequencing; NSCLC: non-small cell lung cancer; PCR: polymerase chain reaction; RCC: renal cell cancer; sqLC: squamous lung cancer; UC: urothelial carcinoma.

**Table 2:** Methods used in clinical trials to identify patients for FGFR inhibitor treatment.

Technology	Pros	Cons	Patient Population *	Prevalence
FGFR protein expression				
Immunohistochemistry	Broadly available, direct measure of receptor expression, keeps spatial resolution, short TAT	No single antibody, needs multiplexing for pan-FGFR inhibitors, Requires pathologist training or central testing	FGFR2b + gastric cancer	30%
**FGFR mRNA expression**				
PCR	Sensitive, cheap, short TAT Easy to establish for each FGFR subtype	No preservation of spatial resolution	FGFR4 + HCC pts (Roblitinib)	Unknown
Nanostring	Sensitive, highly multiplex testing	Expensive, tumor content needs to be retrospectively calculated	FGFR1/2/3 + all comers (Rogaratinib) FGFR2 + gastric cancer (AZD4547)	Up to 25% Unknown
RNA-ISH	Sensitive, keeps spatial resolution, IHC-like workflow, short TAT, multiplex possible	Requires pathologist training or central testing	FGFR1/2/3 + all comers (Rogaratinib) FGFR1&3 + urothelial cancer patients (Rogaratinib)	25%
RNAseq	Sensitive, highly multiplex testing	Expensive, long TAT (several weeks), no preservation of spatial resolution,	Not applied in any FGFR inhibitor trial to date	Unknown
**FGFR DNA alterations**				
FISH	Keeps spatial resolution	Requires fluorescence microscopy, multiplex possible	FGFR2 + gastric cancer (AZD4547)	4–7% [11]
PCR	Short TAT (7 days)	No preservation of spatial resolution	FGFR2&3 fusion and FGFR3 mutations in urothelial carcinoma (QIAGEN’s FDA approved CDx therascreen^®^ FGFR kit for Erdafitinib)	20% [7]
NGS	Highly multiplex testing	Expensive, long TAT, no preservation of spatial resolution	FGFR2 fusion-positive iCCA (Foundation One™ as FDA approved CDx for Pemigatinib & Infigratinib)	10% [91,98]
**FGF ligand**				
IHC	Broadly available, direct measure of receptor expression, keeps spatial resolution, short TAT	No single antibody, needs multiplexing for pan-FGFR inhibitors, Requires pathologist training or central testing	FGF-19 serum levels in HCC (Fisogatinib)	27% [32]

CDx: Companion diagnostics; FDA: Food and Drug Administration; FISH: fluorescence in situ hybridization; HCC: hepatocellular carcinoma; iCCA: intrahepatic cholangiocellular carcinoma: IHC: immunohistochemistry; NGS: next generation sequencing; PCR: polymerase chain reaction; RNA-ISH: RNA in situ hybridization; RNA-seq: RNA sequencing; TAT: turnaround time. * Only patient populations that have been enrolled into FGFR inhibitor trials.

## Data Availability

Not applicable.
